# Exploring the Links Between Coping Strategies, Emotional Intelligence, and Age in Adolescents with Neuromotor Disabilities

**DOI:** 10.3390/children11121466

**Published:** 2024-11-30

**Authors:** Ioana Elena Cioca, Maria Veronica Morcov, Corina Sporea, Oana Alina Apostol, Angelo Pellegrini, Elena-Nicoleta Bordea

**Affiliations:** 1 Faculty of Midwifery and Nursing, University of Medicine and Pharmacy “Carol Davila”, 37 Dionisie Lupu Street, 020021 Bucharest, Romania; ioanaecioca@yahoo.com (I.E.C.); corina.sporea@gmail.com (C.S.); pellegrini.angelo@gmail.com (A.P.); lilianabordea2015@gmail.com (E.-N.B.); 2 National Teaching Center for Children’s Neurorehabilitation “Dr. Nicolae Robanescu”, 44 Dumitru Minca Street, 041408 Bucharest, Romania; apostol.oana.alina@gmail.com

**Keywords:** emotional intelligence, coping strategies, adolescents, neuromotor disabilities

## Abstract

Background/Objectives: The aim of this study is to explore the relationship between emotional intelligence and coping strategies used by adolescents with disabilities, on the one hand to understand how emotional skills influence stress management and everyday challenges and, on the other hand, considering that it could help specialists to develop interventions and educational programs that support the improvement of emotional skills and coping strategies among adolescents with disabilities. Methods: This cross-sectional study included 100 Romanian adolescents with neuromotor disabilities aged 13 to 18 years (M = 15.2) divided into three groups according to the stages of adolescence: Group 1 (13 years—46 respondents), Group 2 (14 to 17 years—26 respondents), and Group 3 (18 years—28 respondents). They completed a questionnaire that assessed the emotional intelligence of the adolescents (positive impression, interpersonal, intrapersonal, stress management, adaptability, and general mood) as well as another one that assessed the possible cognitive emotional coping strategies (e.g., “blame self”, “acceptance”, “rumination”, “positive refocusing”, “refocus on planning”, “positive reappraisal”, “putting into perspective”, “catastrophizing”, and “blaming others”) that they may adopt in managing life situations related to their health problem. Results: Group 3 has significantly higher scores than Group 2 on the Interpersonal subscale and also significantly higher scores than Group 1 on the Stress management. Our analysis also revealed significant correlations between adaptive coping strategies and emotional intelligence domains across all groups, with positive associations between acceptance and stress management, as well as between positive refocusing and adaptability. Regression analysis identified adaptive coping strategies as significant predictors of emotional intelligence, explaining 29% of its variance. Conclusions: Our findings underscore the critical importance of targeted interventions to enhance emotional regulation and adaptive coping strategies in adolescents with neuromotor disabilities. By focusing on strengthening emotional intelligence and tailoring interventions to developmental stages, these programs can promote better emotional and social functioning, particularly in challenging situations.

## 1. Introduction

Emotional intelligence (EI) has attracted substantial scholarly and public interest following its popularization by Goleman’s foundational work, with its theoretical roots traceable to the 19th century [1].

EI pertains to a spectrum of competencies that empower individuals to effectively process and apply emotional information [2].

The construct has been a subject of contention in the scientific community concerning its classification, with some positing it as a facet of personality and others as a type of intelligence. According to the prevailing ability model, EI constitutes a specific intelligence that facilitates adaptive management of emotions, problem solving, and environmental adaptation [3].

In their seminal work, Salovey and Mayer (1990) elucidated EI as the cognitive capacity to perceive, comprehend, and regulate emotions within oneself and others, positing it as a conduit between cognitive and emotional domains [4].

Bar-On further developed this framework with his emotional-social intelligence model, which underscores the integration of emotional and social competencies, such as adaptability and coping mechanisms, as indispensable elements [1]. The coping is a response that reduces the emotional, bodily, and psychological suffering that is associated with difficult life circumstances and everyday annoyances [5].

Despite the well-documented link between EI and stress [6], the interplay between EI and coping strategies remains less clearly understood. While several studies have identified significant correlations between these constructs [7,8,9,10], others have failed to discern a consistent relationship [11,12]. Notably, scholarly inquiry into the intersection of EI and coping strategies in individuals with disabilities is scarce, despite evidence suggesting that children and adolescents with disabilities confront distinct challenges in the recognition and regulation of emotions, often exacerbated by their physical or cognitive constraints.

From the earliest period of a child’s development, social, emotional, intellectual and motor skills continuously improve [13].

Children with disabilities may have physical, cognitive or social limitations, impairing their ability to recognize emotions [14].

Because physical or neurological limitations may make it more difficult for adolescents with neuromotor disabilities to cope with stress, developing EI is essential to helping them learn how to adjust to stress and improve their quality of life [15].

Disability can significantly contribute to anxiety in adolescence and their families, as it often introduces a range of emotional, social, and practical challenges. Adolescents with disabilities may face heightened levels of stress due to physical limitations, social stigma, or difficulties in achieving independence, which are critical aspects of identity development during this life stage. They may also experience anxiety stemming from peer rejection, bullying, or fear of being perceived as different. For families, the presence of a disability in an adolescent often brings additional concerns about their child’s future, social integration, and the adequacy of available support systems. Parents and caregivers may grapple with feelings of guilt, worry, and helplessness while managing the emotional and financial demands of caregiving. This shared experience of anxiety within the family unit underscores the need for holistic interventions that support both the adolescent and their family, addressing not only the physical and developmental needs associated with the disability but also the emotional and psychological wellbeing of everyone involved. Anxiety and emotional intelligence (EI) play critical yet contrasting roles in treatment adherence. Both highlight the importance of emotional factors in treatment adherence, with high EI potentially buffering the negative effects of anxiety, fostering resilience, and supporting better health outcomes. It is well-known that stress is an important factor that affects everyone, and recent challenges such as the pandemic, bullying, migration, and geopolitical instability serve as evidence of this [16,17,18,19,20,21,22,23].

Moreover, several studies [24,25,26,27] found that children’s emotional development and expression are also closely connected to the parenting style practiced within the family. Self-regulation and emotion management techniques, when used, support emotional stability and give them the strength needed to face life’s challenges [13].

Greater attention from social policies toward people with disabilities would lead to improved self-esteem, enhanced support from family, friends, and the school environment and, simultaneously, facilitate the social inclusion of these individuals [28].

The aim of our study was to analyze the relationship between EI and coping strategies used by adolescents with neuromotor disabilities based on the idea that young people with higher levels of EI may be better able to manage emotions and stress, which helps them to choose more adaptive coping strategies.

Our study proposed the following hypotheses:

**H1:**  *The ability to form and maintain satisfying interpersonal relationships increases as adolescents get older.*

**H2:**  *The use of more adaptive strategies increases as adolescents get older.*

**H3:**  *There is a significant positive correlation between the use of adaptive coping strategies and the domains of EI among adolescents.*

**H4:**  *Emotional regulation strategies predict EI.*

## 2. Materials and Methods

This cross-sectional study was conducted between April and October 2024 at a national neurorehabilitation center specializing in neurorehabilitation for children and adolescents with neuromotor disabilities. The center provides multidisciplinary services, including medical, educational, and psychological support, for individuals aged 0 to 18 years. The study included 100 adolescents with neuromotor disabilities, divided into 3 groups according to the stages of adolescence [29] but adapted at the same time to the requirements of the Cognitive Emotion Regulation questionnaire, which stipulates a lower age limit of administration to the age of 13 years. Also, the maximum age for admission to the center is 18 years. Thus, the following groups were obtained: Group 1 (13 years—46 respondents), Group 2 (14 to 17 years—26 respondents), Group 3 (18—28 respondents).

The participants were recruited using purposive sampling. The inclusion criteria were as follows: a diagnosis of neuromotor disability, age between 13 and 18 years, and an IQ score of 70 or above. All participants provided informed consent, and the study was approved by the Ethics Committee of the neurorehabilitation center (protocol code 6057, approval date 19 April 2024). To ensure participant confidentiality, all personal data were anonymized. The study adhered to the ethical principles outlined in the Declaration of Helsinki, ensuring that participants’ rights were respected throughout the research process.

The level of emotional and social functioning was measured using Romanian version of the Emotional Quotient Inventory: Youth Version (EQ-i:YV™) [30], a widely recognized assessment questionnaire containing 60 items. The questionnaire is organized into 7 dimensions as follows [21]: Total EQ, Interpersonal, Intrapersonal, Adaptability, Stress Management, General Mood, and Positive Impression. A 4-point Likert scale, ranging from 1 (“very seldom true or not true for me”) to 4 (“very often true of me or true of me”) was used to record responses to each item [31].

A score greater than 130 suggests the presence of extremely well-developed emotional and social skills while a score below 70 refers to the presence of severely underdeveloped emotional and social abilities, highlighting the importance of focusing on improving and growing such skills [32]. 

Cronbach’s Alpha ranged from a = 0.72 for the Positive Impression dimension to a = 0.88 for the General Mood in the masculine’s sample, while internal consistency ranged from a = 0.69 for the Positive Impression dimension to a = 0.89 for the General Mood in the feminine’ s sample.

The conscious cognitive strategies of emotion regulation were analyzed using Cognitive Emotion Regulation Questionnaire (CERQ), a five-point Likert scale that ranged from one (almost never) to five (almost always) [33,34,35]. Nine subscales (with 4 items each) include the following strategies: “Blame self”, “Acceptance”, “Rumination”, “Positive refocusing”, “Refocus on planning”, “Positive reappraisal”, “Putting into perspective”, “Catastrophizing”, and “Blaming others” [36]. These strategies could be “adaptive” or “maladaptive” [36,37]. Greater use of the strategy is reflected in higher scores.

Cronbach’s Alpha ranged from a = 0.68 for the blaming others to a = 0.83 for the rumination strategy [38].

Respondents’ intelligence coefficient (IQ) was measured using the Standard Progressive Matrice (SPM), which is an assessment tool designed for evaluating intelligence in individuals aged 6 to 80 years [39]. SPM has five series of 12 items each, and the complexity of the items increases with each series. Every diagram has a missing piece, which the subject has to find from the given response choices [40]. A score between 70 and 80 represents a liminal intelligence and a score over 80 means normal intelligence [40].

All analyses were performed with Microsoft Excel and SPSS—version 22—soft. Descriptive statistics were used to summarize the socio-demographic characteristics, while inferential statistics, such as the Kruskal–Wallis test, Spearman’s rho correlation, and linear regression, were used to examine the relationships between age, coping strategies, and emotional intelligence.

## 3. Results

The socio-demographic characteristics of the participants, including gender, age, residence, and cognitive functioning, are summarized in Table 1 below. The mean age of the participants was 15.2 years.

An analysis was conducted to examine the distribution of the data, and it was discovered that the data did not follow a normal distribution (*p* < 0.05).

To determine how the scores for each group were distributed, we calculated the EQ-i:YV™ questionnaire’s median and standard deviation. 

According to the results, the groups are similar in medians on most EQ-i:YV™ subscales; however, Group 3 tends to have higher median scores on Positive Impression and Stress Management (Table 2).

We used the Kruskal–Wallis test on each subscale to search for significant differences between groups, even when the medians were equal. This non-parametric test identifies possible differences in the distributions of group scores even when measures of central tendency, such as the median, are similar.

The Kruskal–Wallis test (Table 3) revealed that the difference was statistically significant at *p* < 0.05 (*p* = 0.031 for interpersonal and *p* = 0.003 for stress management).

Post hoc analysis using Tukey’s HSD was conducted for the variables exhibiting statistically significant differences to identify the specific groups where these differences were significant.

As shown in Table 4, Group 3 has significantly higher scores than Group 2 (*p* = 0.030) on the Interpersonal subscale and also significantly higher scores than Group 1 (*p* = 0.003) on the Stress management.

We also calculated the median and standard deviation of the CERQ to figure out the distribution of scores for each group. According to the results in Table 4, Group 1 showed higher medians in comparison to Groups 2 and 3 for maladaptive coping strategies, including Blame self, Rumination, and Blaming others, indicating an increased tendency for this group to use these strategies compared to the others. 

Group 2 has higher medians compared to Groups 1 and 3 across all adaptive coping strategies, including Acceptance, Positive refocusing, Refocus on planning, Positive reappraisal, and Putting into perspective. This suggests that Group 2 uses these strategies more frequently than the other groups (Table 5).

Furthermore, we evaluated whether the differences in medians among the groups were statistically significant using the Kruskal–Wallis test.

The Kruskal–Wallis test (Table 6) revealed that the difference was statistically significant at *p* < 0.05 (*p* = 0.003 for acceptance, *p* = 0.007 for positive reappraisal, *p* = 0.015 for putting into perspective, and *p* = 0.011 for catastrophizing).

The post hoc analysis indicated that Group 1 had a higher mean difference for acceptance (*p* = 0.003), positive reappraisal (*p* = 0.007), and catastrophizing (*p* = 0.009) than Group 3 (Table 7).

An analysis of the relationship between the variables measured by the CERQ and the EQ-i:YV™ was carried out in order to determine whether or not emotional regulation strategies are associated with the level of EI.

Consequently, Spearman’s correlation coefficient was used to examine relationships between different dimensions of cognitive-emotional coping and EI for each group (Table 8).

In the analysis of Group 1 characteristics, weak to moderate, statistically significant positive correlations were observed between all adaptive coping strategies and some EI domains. Our findings included correlations between Acceptance and Stress management (ρ = 0.302) and Total EQ (ρ = 0.312), as well as Positive refocusing and Adaptability (ρ = 0.355).

Additionally, Refocus on planning was positively associated with Positive impression (ρ = 0.511), Intrapersonal (ρ = 0.373), and Adaptability (ρ = 0.293).

Positive reappraisal demonstrated correlations with Positive impression (ρ = 0.415) and Intrapersonal (ρ = 0.311), while Putting into perspective was corelated to Positive impression (ρ = 0.296).

Moreover, two moderate statistically significant positive correlations were identified between a maladaptive coping strategy, Rumination and both Intrapersonal (ρ = 0.518), and Total EQ (ρ = 0.483).

In Group 2, we found moderate to high, statistically significant positive and negative correlations between several maladaptive coping strategies and certain domains of emotional intelligence. Thus, Blame self was associated with Positive impression (ρ = 0.414) and Adaptability (ρ = 0.618), Rumination with Adaptability (ρ = 0.478), and Catastrophizing with Stress management (ρ = −0.404).

Among adaptive coping strategies, three negative and three positive, moderate statistically significant correlations were identified with EI domains. 

Positive refocusing was negatively corelated with Intrapersonal (ρ = −0.557), General mood (ρ = −0.517), and Total EQ (ρ = −0.524).

Refocus on planning correlated was positively correlated with Adaptability (ρ = 0.674), while Putting into perspective with Intrapersonal (ρ = 0.467) and Total EQ (ρ = 0.420).

When analyzing the characteristics in Group 3, moderate to high statistically significant positive and negative correlations were found between adaptive coping strategies and various domains of EI, with three of the five adaptive strategies showing consistent positive associations with domains of EI. Thus, Refocus on planning was positively associated with Adaptability (ρ = 0.441), Positive reappraisal with Positive impression (ρ = 0.424), Adaptability (ρ = 0.432), and General mood (ρ = 0.575). At the same time, Positive refocusing was negatively corelated with Stress management ρ = −0.512).

Moreover, we identified moderate to high statistically significant positive and negative correlations between all maladaptive coping strategies and certain domains of EI: Blame self with Intrapersonal (ρ = 0.504), Rumination with both Intrapersonal (ρ = 0.520), and Total EQ (ρ = 0.432), Catastrophizing with Stress management (ρ = −0.586), and Blaming others with Stress management (ρ = −0.685) and Total EQ (ρ = −0.396).

To investigate the extent to which coping strategies influence the Total EQ, we conducted a linear regression analysis (Table 9). This analysis allowed us to assess the specific contribution of each coping strategy to the total EI score.

Model summary: R: 0.539, R^2^: 0.290, Adjusted R^2^: 0.219, F (df = 9, 90): 4.090, Sig.: 0.000.

The linear regression analysis reveals that specific coping strategies significantly predict Total Emotional Intelligence (EQ). The model explains 29% of the variance in Total EQ (R^2^ = 0.290), with an adjusted R^2^ of 0.219. The overall model is statistically significant (F = 4.090, *p* < 0.001). Among the coping strategies analyzed, Rumination (*p* = 0.034) and Putting into perspective (*p* = 0.009) positively predict Total EQ, indicating that these strategies are associated with higher emotional intelligence. Conversely, Positive refocusing (*p* = 0.021) and Catastrophizing (*p* = 0.041) negatively predict Total EQ, suggesting that maladaptive strategies like catastrophizing hinder emotional intelligence. Other strategies, such as Self-blame, Acceptance, and Refocus on planning did not show significant relationships with Total EQ in this model (Table 9).

## 4. Discussion

Exploring the relationship between emotional intelligence (EI) and coping strategies in adolescents with neurodisabilities is crucial because it provides insights into how these individuals navigate the unique emotional and social challenges they face. 

The purpose of our study was to assess the relationship between coping strategies and EI of adolescents with neuromotor disabilities.

By understanding how EI influences the choice and effectiveness of coping strategies, researchers and practitioners can identify pathways to strengthen resilience and emotional wellbeing in this population.

Our analysis has demonstrated that there is a significant difference in the ability to form and maintain satisfactory interpersonal relationships among the three studied groups. The results obtained indicate a clear hierarchy in the performance of the groups, with Group 3 having the highest capacity, followed by Group 2 and then Group 1. This finding confirms our first hypothesis, which assumed that interpersonal relationship increases as adolescents get older.

Our results are in agreement with a study in the literature that appreciates the importance of EI in adolescent development with impact on effective social interaction [41].

The analyzed data do not confirm the hypothesis that as adolescents grow older, they increasingly use adaptive coping strategies, as there is no progressive increase in the use of adaptive strategies as adolescents age. The order of preferences for the adaptive strategies Acceptance and Positive Reappraisal shows that adolescents in Groups 1 and 2 (i.e., younger ones) use these strategies more than those in Group 3. If the hypothesis had been confirmed, we would have expected Group 3 (older adolescents) to adopt adaptive strategies significantly more often than the younger groups. Regarding Putting into perspective, Group 2 uses it more than the other groups. Therefore, rather, we could appreciate that the distribution of the use of adaptive strategies varies between groups, without speaking of a clear trend of increase proportional to the age of adolescents, suggesting that this relationship could be influenced by factors that could be further analyzed.

According to the findings, Group 1 showed the highest pattern of positive relationships between adaptive strategies for coping and EI categories. For each adaptive strategy, Group 1 revealed statistically significant positive associations with various domains of EI, such as Positive impression, Intrapersonal, Stress management, Adaptability, and Total EQ, confirming our third hypothesis for this group.

Groups 2 and 3, on the other hand, demonstrate both positive and negative relationships between adaptive strategies and EI domains. As an example, Positive refocusing has a negative correlation with Intrapersonal, General mood, and Total EQ in Group 2 and a negative correlation with Stress management in Group 3. These findings indicate that, although EI domains and adaptive coping strategies are positively correlated, our third hypothesis is not entirely supported for these two groups.

Regression analysis highlights how different coping strategies may affect EI. Data from the linear regression analysis support our fourth hypothesis; Total EQ is predicted by coping strategies, particularly by adaptive ones

Overall, results from the literature indicate a predictive relationship between emotional regulation strategies and EI [6,10], with the trend toward the consensus that emotional coping strategies contribute positively to the development and enhancement of EI. Therefore, we can reasonably support our fourth hypothesis. 

Our study found that rumination—typically considered a maladaptive coping strategy—was positively associated with Total EQ, which may seem counterintuitive. While rumination is generally linked to negative outcomes, this finding could reflect a more nuanced relationship. It is possible that adolescents who engage in rumination might process their emotions more deeply, potentially leading to greater emotional awareness and, consequently, higher EI. This points to the complexity of emotional regulation and highlights the need for further exploration of the role of rumination in emotional development.

Nonetheless, this could indicate a complex relationship where individuals who ruminate might engage in deeper emotional processing, potentially enhancing their emotional awareness.

Integrating EI training into school curricula can equip adolescents with neurodisabilities with the tools to manage stress, build positive relationships, and navigate social situations more effectively. Similarly, therapeutic interventions focusing on enhancing EI may empower these individuals to adopt adaptive coping strategies, such as seeking social support or problem solving, rather than maladaptive ones like avoidance or withdrawal. On a policy level, understanding the interplay between EI and coping could lead to the creation of more inclusive educational environments that prioritize social–emotional learning, foster peer acceptance, and reduce stigma.

### Limitations

Our study has several limitations that should be considered when interpreting the findings. The sample size, though adequate for preliminary analysis, was relatively small, which may limit the generalizability of the results and the statistical power of some of our tests. A larger, more diverse sample would provide more robust data and allow for more definitive conclusions. Additionally, the cross-sectional design of the study means that causal relationships cannot be inferred. Longitudinal studies would be necessary to examine how coping strategies and EI evolve over time and to better understand the directionality of their relationship.

Another limitation is the lack of consideration of other factors that may influence both EI and coping strategies, such as family support, social relationships, and gender differences. These factors could have a significant impact on emotional regulation and coping mechanisms, and future research should aim to explore how these variables interact with EI in adolescents with neuromotor disabilities. Furthermore, the study focused exclusively on adolescents attending a specialized rehabilitation center, which may limit the applicability of the findings to other populations of adolescents with neuromotor disabilities, particularly those not receiving structured interventions.

## 5. Conclusions

Despite the limitations, our study has important practical implications. The findings suggest that interventions aimed at improving adaptive coping strategies could help enhance EI in adolescents with neuromotor disabilities. Given the significant role of emotional regulation in social interaction, academic performance, and overall wellbeing, enhancing EI could have broad benefits for adolescents in this population. Specifically, targeting coping strategies such as Acceptance, Positive Reappraisal, and Positive Refocusing may help adolescents improve their emotional and social functioning, particularly in stressful situations.

Moreover, the variability observed in the use of adaptive strategies across different age groups highlights the importance of tailoring interventions to the specific developmental stage of each adolescent. For younger adolescents, focusing on strengthening adaptive coping strategies and providing emotional support might be particularly beneficial, while for older adolescents, interventions could focus on refining existing coping mechanisms and promoting emotional resilience. 

Exploring relationships between EI and copying strategies addresses both the emotional and social dimensions of living with a neurodisability, promoting not only individual wellbeing but also greater societal inclusion. This research can pave the way for evidence-based programs that improve quality of life and long-term outcomes for adolescents with neurodisabilities.

## Figures and Tables

**Table 1:** Socio-demographic characteristics of the participants.

Variable	Frequency	Percent
Gender		
Male	52	52.0
Female	48	48.0
Age		
13 years	46	46.0
14–17 years	26	26.0
18 years	28	28.0
Residence		
Urban	76	76.0
Rural	24	24.0
QI		
70–80 (borderline)	33	33.0
>80 (normal)	67	67.0
Educational status		
Currently in School	100	100.0
Total	100	100.0

**Table 2:** Descriptive statistics for EQ-i:YV™ for three groups.

EQ-i:YV™	Group 1		Group 2		Group 3	
	Median	Std. Dev.	Median	Std. Dev.	Median	Std. Dev.
Positive impression	4.0	0.756	4.0	1.265	5.0	0.989
Interpersonal	4.0	0.926	4.0	1.087	4.0	0.826
Intrapersonal	4.0	1.068	4.0	1.359	4.0	0.976
Stress management	4.0	1.226	4.0	1.104	5.0	1.145
Adaptability	4.0	0.786	4.0	1.442	4.0	0.976
General mood	4.0	0.774	4.0	1.386	4.0	0.9
Total EQ	4.0	1.201	4.0	1.327	4.0	0.932

**Table 3:** Kruskal–Wallis computations on EQ-i:YV™ and age groups.

EQ-i:YV™		Sum of Squares	df	Mean Square	F	Sig.
Positive impression	Between Groups	5.592	2	2.796	2.943	0.057
	Within Groups	92.168	97	0.950		
	Total	97.760	99			
Interpersonal	Between Groups	6.424	2	3.212	3.599	0.031
	Within Groups	86.576	97	0.893		
	Total	93.000	99			
Intrapersonal	Between Groups	0.388	2	0.194	0.153	0.859
	Within Groups	123.172	97	1.270		
	Total	123.560	99			
Stress management	Between Groups	16.898	2	8.449	6.137	0.003
	Within Groups	133.542	97	1.377		
	Total	150.440	99			
Adaptability	Between Groups	1.220	2	0.610	0.560	0.573
	Within Groups	105.540	97	1.088		
	Total	106.760	99			
General mood	Between Groups	4.626	2	2.313	2.318	0.104
	Within Groups	96.814	97	0.998		
	Total	101.440	99			
Total EQ	Between Groups	3.062	2	1.531	1.122	0.330
	Within Groups	132.298	97	1.364		
	Total	135.360	99			

**Table 4:** The post hoc analysis of various EI domains by age group.

Dependent Variable	(I) Age Group	(J) Age Group	Mean Difference (I-J)	Std. Error	Sig.
Interpersonal	Group 1	Group 2	0.482	0.232	0.100
		Group 3	−0.183	0.226	0.698
	Group 2	Group 1	−0.482	0.232	0.100
		Group 3	−0.665 *	0.257	0.030
	Group 3	Group 1	0.183	0.226	0.698
		Group 2	0.665 *	0.257	0.030
Stress management	Group 1	Group 2	−0.625	0.288	0.081
		Group 3	−0.944 *	0.281	0.003
	Group 2	Group 1	0.625	0.288	0.081
		Group 3	−0.319	0.320	0.580
	Group 3	Group 1	0.944 *	0.281	0.003
		Group 2	0.319	0.320	0.580

* Correlation is significant at the 0.05 level.

**Table 5:** Descriptive statistics for CERQ for three groups.

CERQ	Group 1		Group 2		Group 3	
	Median	Std. Dev.	Median	Std. Dev.	Median	Std. Dev.
Blame self	7.0	2.041	5.0	1.098	5.0	1.864
Acceptance	6.0	1.387	7.0	1.806	4.5	1.526
Rumination	7.0	1.445	6.0	0.992	6.0	1.462
Positive refocusing	6.0	1.761	7.0	1.85	5.0	2.073
Refocus on planning	6.0	1.163	7.0	1.164	6.0	1.569
Positive reappraisal	6.0	1.584	7.0	1.813	5.0	1.989
Putting into perspective	5.0	1.513	7.0	1.521	5.0	1.782
Catastrophizing	7.0	0.888	7.0	1.497	6.0	1.193
Blaming others	7.0	2.264	6.0	2.046	4.5	1.484

**Table 6:** Kruskal–Wallis computations on coping strategies and age groups.

		Sum of Squares	df	Mean Square	F	Sig.
Blame self	Between Groups	7.551	2	3.775	1.176	0.313
	Within Groups	311.489	97	3.211		
	Total	319.040	99			
Acceptance	Between Groups	29.636	2	14.818	6.222	0.003
	Within Groups	231.004	97	2.381		
	Total	260.640	99			
Rumination	Between Groups	1.197	2	0.599	0.329	0.720
	Within Groups	176.243	97	1.817		
	Total	177.440	99			
Positive refocusing	Between Groups	7.343	2	3.672	1.044	0.356
	Within Groups	341.017	97	3.516		
	Total	348.360	99			
Refocus on planning	Between Groups	2.856	2	1.428	0.859	0.427
	Within Groups	161.144	97	1.661		
	Total	164.000	99			
Positive reappraisal	Between Groups	32.559	2	16.280	5.231	0.007
	Within Groups	301.881	97	3.112		
	Total	334.440	99			
Putting into perspective	Between Groups	22.123	2	11.062	4.353	0.015
	Within Groups	246.517	97	2.541		
	Total	268.640	99			
Catastrophizing	Between Groups	12.653	2	6.327	4.724	0.011
	Within Groups	129.907	97	1.339		
	Total	142.560	99			
Blaming others	Between Groups	3.307	2	1.654	0.406	0.667
	Within Groups	394.653	97	4.069		
	Total	397.960	99			

**Table 7:** The post hoc analysis of multiple coping strategies by age group.

Dependent Variable	(I) Age Group	(J) Age Group	Mean Difference (I-J)	Std. Error	Sig.
Acceptance	Group 1	Group 2	0.134	0.379	0.934
		Group 3	1.255 *	0.370	0.003
	Group 2	Group 1	−0.134	0.379	0.934
		Group 3	1.121 *	0.420	0.024
	Group 3	Group 1	−1.255 *	0.370	0.003
		Group 2	−1.121 *	0.420	0.024
Positive reappraisal	Group 1	Group 2	0.124	0.433	0.956
		Group 3	1.311 *	0.423	0.007
	Group 2	Group 1	−0.124	0.433	0.956
		Group 3	1.187 *	0.480	0.040
	Group 3	Group 1	−1.311 *	0.423	0.007
		Group 2	−1.187 *	0.480	0.040
Putting into perspective	Group 1	Group 2	−0.314	0.391	0.702
		Group 3	0.894	0.382	0.055
	Group 2	Group 1	0.314	0.391	0.702
		Group 3	1.209 *	0.434	0.018
	Group 3	Group 1	−0.894	0.382	0.055
		Group 2	−1.209 *	0.434	0.018
Catastrophizing	Group 1	Group 2	0.478	0.284	0.216
		Group 3	0.835 *	0.277	0.009
	Group 2	Group 1	−0.478	0.284	0.216
		Group 3	0.357	0.315	0.496
	Group 3	Group 1	−0.835 *	0.277	0.009
		Group 2	−0.357	0.315	0.496

* The mean difference is significant at the 0.05 level.

**Table 8:** Spearman’s rho correlations between CERQ and EQ-i:YV™ subscales for each group.

Correlations									
			Positive Impression	Interpersonal	Intrapersonal	Stress Management	Adaptability	General Mood	Total EQ
Group 1	Spearman’s rho	Blame self	0.013	0.143	0.328 *	−0.306 *	−0.052	0.023	0.204
		Acceptance	0.174	0.082	0.262	0.302 *	−0.044	0.046	0.312 *
		Rumination	0.278	0.132	0.518 **	0.138	0.288	0.092	0.483 **
		Positive refocusing	0.233	0.052	0.235	0.151	0.355 *	−0.005	0.122
		Refocus on planning	0.511 **	0.112	0.373 *	0.211	0.293 *	0.181	0.288
		Positive reappraisal	0.415 **	−0.003	0.311 *	0.051	0.279	0.277	0.135
		Putting into perspective	0.296 *	0.174	0.197	0.151	0.131	0.142	0.264
		Catastrophizing	0.205	0.217	0.103	−0.032	00.000	0.147	0.281
		Blaming others	0.068	0.208	0.242	−0.222	−0.030	0.133	0.257
Group 2	Spearman’s rho	Blame self	0.414 *	−0.079	−0.033	0.045	0.618 **	0.299	0.027
		Acceptance	−0.076	−0.072	−0.195	−0.319	0.308	−0.024	−0.114
		Rumination	0.037	−0.057	−0.227	0.059	0.478 *	−0.227	−0.121
		Positive refocusing	−0.277	−0.057	−0.557 **	−0.299	−0.255	−0.517 **	−0.524 **
		Refocus on planning	−0.168	0.078	−0.036	−0.098	0.674 **	0.177	0.040
		Positive reappraisal	0.111	−0.311	0.153	0.126	−0.171	0.064	0.106
		Putting into perspective	0.186	−0.035	0.467 *	0.229	−0.044	0.158	0.420 *
		Catastrophizing	−0.125	0.036	0.045	−0.404 *	0.237	−0.160	−0.166
		Blaming others	−0.026	−0.193	0.164	−0.327	−0.180	−0.115	−0.119
Group 3	Spearman’s rho	Blame self	−0.008	0.061	0.504 **	0.228	−0.073	0.317	0.349
		Acceptance	0.063	0.079	00.000	0.057	0.161	0.109	0.111
		Rumination	−0.363	0.334	0.520 **	−0.225	−0.063	0.318	0.432 *
		Positive refocusing	0.333	0.212	0.134	−0.512 **	0.317	0.359	−0.036
		Refocus on planning	0.199	0.227	0.209	−0.254	0.441 *	0.233	0.116
		Positive reappraisal	0.547 **	0.361	0.263	−0.237	0.582 **	0.637 **	0.248
		Putting into perspective	0.307	0.424 *	0.359	−0.273	0.432*	0.575 **	0.278
		Catastrophizing	−0.313	0.042	0.053	−0.586 **	−0.158	−0.249	−0.261
		Blaming others	−0.138	0.146	−0.043	−0.685 **	−0.215	−0.251	−0.396 *

** Correlation is significant at the 0.01 level (two-tailed). * Correlation is significant at the 0.05 level (two-tailed).

**Table 9:** Linear regression analysis of coping strategies.

Coefficients ^a^						
Model		Unstandardized Coefficients		Standardized Coefficients	t	Sig.
		B	Std. Error	Beta		
1	(Constant)	81.390	9.208		8.840	0.000
	Blame self	−0.359	0.987	−0.044	−0.364	0.716
	Acceptance	−0.477	1.043	−0.053	−0.457	0.648
	Rumination	3.065	1.427	0.279	2.148	0.034
	Positive refocusing	−1.964	0.838	−0.251	−2.344	0.021
	Refocus on planning	2.181	1.474	0.191	1.479	0.143
	Positive reappraisal	0.398	1.119	0.050	0.356	0.723
	Putting into perspective	2.857	1.063	0.320	2.688	0.009
	Catastrophizing	−3.470	1.676	−0.283	−2.070	0.041
	Blaming others	0.926	1.014	0.126	0.913	0.364

^a^ Dependent Variable: Total EQ.

## Data Availability

The corresponding authors can provide the data described in this study upon request. The data are not publicly available due to privacy restrictions.
